# Where do we go from here? Reconciling implementation failure of PrEP for Black women in the South. Leveraging critical realism to identify unaddressed barriers as we move forward

**DOI:** 10.3389/frph.2024.1449554

**Published:** 2024-11-19

**Authors:** Whitney C. Irie, Anais Mahone, Renee Heffron, Latesha Elopre

**Affiliations:** ^1^ School of Social Work, Boston College, Chestnut Hill, MA, United States; ^2^ Department of Medicine, University of Alabama at Birmingham, Birmingham, AL, United States

**Keywords:** Black women, PrEP (pre-exposure prophylaxis) for HIV prevention, critical realism, implementation science, Southern United States (U.S.)

## Abstract

**Introduction:**

PrEP, a highly effective HIV prevention measure, provides autonomy to individuals in managing their HIV acquisition vulnerability. Despite its availability in tenofovir-based oral pills and injectable cabotegravir formulations, PrEP uptake among Black cisgender women in the U.S. South, a region with a high HIV burden, remains critically low. This demographic faces a disproportionately high rate of new HIV diagnoses, yet fewer than 10% of women in the US who could benefit from PrEP are currently receiving it.

**Methods:**

Utilizing a critical realism interpretative framework, this narrative review employed a tri-level analysis strategy to examine the empirical, actual, and real domains influencing PrEP implementation among Black women in the Southern U.S. The empirical level refers to observable events and data (e.g., PrEP uptake rates), the actual level encompasses experiences and actions that may not always be directly observed (e.g., healthcare interactions and community engagement), and the real level involves the deeper structures and mechanisms (e.g., systemic racism and cultural narratives) that shape these outcomes. A comprehensive search of peer-reviewed literature from PubMed and other sources was conducted to identify barriers and facilitators to PrEP uptake in this population.

**Results:**

The analysis revealed significant barriers, including structural violence, socioeconomic disparities, medical mistrust, stigma, and inadequate healthcare policies. Empirical data showed variability in PrEP awareness and interest among Black women, while actual experiences highlighted misaligned marketing strategies, financial constraints, and interpersonal dynamics. At the real level, underlying mechanisms such as systemic racism and cultural narratives were identified as critical impediments to PrEP uptake.

**Discussion:**

Addressing these multifaceted barriers requires a comprehensive, multi-level approach that integrates personalized, community-centric strategies. Emphasizing the need for healthcare providers, community leaders, researchers, and policymakers to collaborate, the review proposes actionable strategies to enhance PrEP implementation, focusing on education, structural reforms, and policy changes to improve access and acceptability among Black women in the South.

## Introduction

In the landscape of HIV prevention, PrEP stands as a cornerstone intervention due to its high effectiveness and the autonomy it provides to individuals in managing their risk of HIV acquisition. The two primary PrEP modalities currently available for cisgender women in the U.S. are tenofovir-based once-daily oral pills and a 2-monthly injectable cabotegravir formulation; both require relatively frequent clinic appointments to receive HIV testing and other corresponding laboratory tests (1, 2). Despite the availability of these modalities, the uptake of PrEP among Black women in the U.S. South, the U.S. region with greatest burden of HIV, is disconcertingly low. Statistically, Black women in this region are disproportionately affected by HIV, accounting for the majority of new HIV diagnoses among women at a rate that is nearly 20 times that of their white counterparts (3). However, recent statistics indicate that fewer than 10% of Black women in the U.S., who could benefit from PrEP are currently receiving it, a stark contrast to the overall need (4).

### Implementation failure of PrEP – understanding the context

The “implementation failure” of PrEP, particularly in the context of Black women in the South, describes a significant gap between the identified need for PrEP and its actual utilization by this population. Despite a clear recognition of the disproportionate HIV incidence in this demographic and PrEP's established efficacy as a preventive measure, the PrEP-to-need ratio (PNR) remains relatively lower than other groups defined by race and gender (5, 6). This chasm indicates that existing efforts to scale up PrEP access have not yet sufficiently resonated with or reached this community. While persistent, initiatives to enhance the PNR have not yielded substantial changes in uptake rates, suggesting a misalignment between the strategies employed and the determinants of health behavior in this population.

Stigma and discrimination related to HIV, sexual behavior, and drug use—heightened by racial and gender biases—create an environment where open discussions and acceptance of PrEP are hindered (7–11). Cultural and religious values further influence these attitudes, discouraging sexual health education and the use of sexual health interventions (12, 13). Moreover, the South's slower adoption of healthcare policies and Medicaid expansion has restricted access to necessary services. These factors, rooted in a history of marginalization, weave a complex web of challenges that Southern Black communities must navigate to improve the implementation of PrEP and other health interventions (14–17).

Public policies serve as critical determinants of PrEP accessibility, especially when addressing structural determinants such as discrimination and stigma. Collectively, these factors significantly influence biomedical intervention access and acceptability among historically marginalized groups in the U.S., including cisgender Black women. Although Medicaid expansion has improved PrEP accessibility for residents in participating states, individuals who are uninsured or face copays, coinsurance, or deductible costs may still encounter barriers to medication uptake. PrEP drug assistance programs (PrEP-DAP) help to narrow these gaps, increasing PrEP prevalence in participating states (18). Notably, states that could benefit most from these programs, often located in the Southern United States, have not implemented Medicaid Expansion or PrEP DAP programs, thereby actively contributing to the widening disparities in PrEP uptake.

Additionally, outreach strategies such as PrEP advertisements and messaging often fail to resonate with Black women. This failure stems from their exclusion in early PrEP campaigns, which primarily targeted men who have sex with men. Despite recent efforts to create more inclusive campaigns, the prevailing perception still largely views PrEP as a preventive measure for this group alone, hindering broader acceptance and uptake among Black women (19). Moreover, even well-intentioned healthcare providers may lack the necessary training or resources to recommend PrEP confidently and effectively, especially to those Black women who stand to benefit the most from it (20). Consequently, practical barriers, such as unawareness and the dearth of culturally competent providers, obstruct its effective delivery and uptake. Current strategies often fall short in tackling these complex issues effectively.

To address these issues, a concerted, multi-level approach is imperative—one that involves a critical evaluation and incorporation of the mechanisms of change and action that are pertinent to PrEP implementation. Such mechanisms need to be identified, understood, critically evaluated, and applied to ensure that strategies are both precise and effective. This calls for a departure from a generalized method to a more personalized, community-centric strategy, one that fully acknowledges and actively addresses the structural and social determinants of health that so markedly impact Black women in the South. Only by embracing such a comprehensive, informed, and empathetic approach can the implementation of PrEP shift from a theoretical public health potential to a tangible success story in these communities.

This article aims to comprehensively assess the current state of PrEP implementation for Black cisgender women in the South by employing a critical realism interpretative framework. To provide a foundational context for this examination, first, we introduce critical realism as a relevant meta-theory within the realm of health and social research and expound upon key implications of critical realism in the field of PrEP among Black women, providing practical examples of how it can be effectively applied to elucidate the causes of shortcomings in PrEP implementation; we then offer a tri-level analysis and review of current studies to identify the factors influencing PrEP implementation in Black women in the South. Finally, we propose a path forward to enhance PrEP implementation strategies among Black women that specifically overcome the challenges identified.

## Theoretical framework

### Getting real: critical realism to improve PrEP implementation among Black women

In the realm of HIV research, there is a pressing need to leverage robust theoretical frameworks capable of accommodating the intricate nature of the HIV continuum. Such a framework should encourage us to adopt a more holistic approach, allowing us to investigate the phenomena of HIV prevention, care, and treatment from various angles and critical perspectives. The work of Bhaskar (1997), a distinguished British sociologist and philosopher, paved the way for such an approach in the 1970s when he introduced Critical Realism as an ontological and epistemological standpoint for exploring the lives of individuals within their social and health contexts (21).

Critical realism offers an interpretive framework that is highly pertinent to studying complex health interventions, like implementing PrEP among Black women in the Southern United States. This philosophical approach compels us to delve beyond the surface of observable phenomena to understand the intricate layers of reality that influence health outcomes (22, 23). It encourages us to consider how personal agency is interwoven with broader structural conditions and cultural influences, all of which coalesce to impact the uptake of health services. Specifically, in the case of PrEP implementation, critical realism prompts us to interrogate not only the glaring disparities in healthcare delivery and utilization but to expose the hidden mechanisms underpinning these inequities (22–24).

By applying a critical realist lens, we acknowledge that the low uptake of PrEP is not merely a consequence of individual choices or isolated barriers but is rooted in a constellation of interdependent factors. These include historical contexts of systemic racism that shape healthcare delivery, socioeconomic conditions limiting access to medical innovation, and cultural narratives influencing health behavior and perceptions. It enables a holistic examination of how social structures, power dynamics, and ideologies contribute to the persistent underutilization of PrEP by Black women in the South.

Moreover, critical realism demands that a clear understanding of these underlying dynamics inform interventions. It advocates for strategies that are not just theoretically sound but are grounded in the actual experiences and needs of Black women in the South. For instance, incorporating critical realism would suggest that PrEP implementation strategies must be multifaceted, addressing not just the dissemination of information but also the transformation of societal attitudes, enhancement of healthcare systems, and empowerment of Black women as informed healthcare participants.

Thus, through the lens of critical realism, we can better design and evaluate interventions by ensuring they are tailored to the identified barriers and informed by an understanding of the causal powers – both seen and unseen – that sustain those barriers (24). This depth of insight can transform PrEP from a mere possibility to an accessible and utilized resource, ultimately narrowing the implementation gap and advancing the health equity for Black women in the South.

## Methods and analysis

### Layering the barriers: narrative review

#### Searches

To ascertain barriers to PrEP uptake, specifically among Black women more likely to be diagnosed with HIV in the Southern United States, we scrutinized peer-reviewed papers and conference abstracts. Our search encompassed PubMed abstracts and articles from 2016 onward. We crafted our search using a combination of terms and phrases that would yield the most pertinent results: (“PrEP” OR “pre-exposure prophylaxis”) AND (“HIV”) AND (“Black women” OR “African American women”) AND (“Southern United States” OR “South” OR specific states like “Georgia” OR “Mississippi” OR “Louisiana”), along with various study types to encapsulate a breadth of research methodologies. We further refined our research during manuscript development by employing these search parameters in PubMed with additional filters for study type and specific barriers such as knowledge, awareness, risk perception, stigma, bias, mistrust, access, cost, side effects, and medication interactions. Additional pertinent references were extracted from the bibliographies of selected articles or discovered through follow-up PubMed searches, ensuring a comprehensive capture of the current landscape of PrEP implementation challenges faced by this demographic.

### Tri-level analysis strategy

Critical realism asserts the importance of distinguishing between the empirical, the actual, and the real in understanding social phenomena (21, 25). This approach is relevant to analyzing PrEP implementation for Black women in the South. At the empirical level, we focus on the observable data—this includes the number of Black women using PrEP, their reported reasons for uptake or non-uptake, and the statistical trends of HIV incidence within their communities. We can measure or document these experiences, behaviors, and events.

At the actual level, we consider the full range of events related to PrEP implementation, whether observed or not. This includes occurrences such as Black women being offered PrEP and declining it, missed opportunities for healthcare interventions, or interactions with healthcare providers that could influence decisions about PrEP. The actual level captures everything that happens, even if it isn't always visible or recorded.

**Figure 1:**
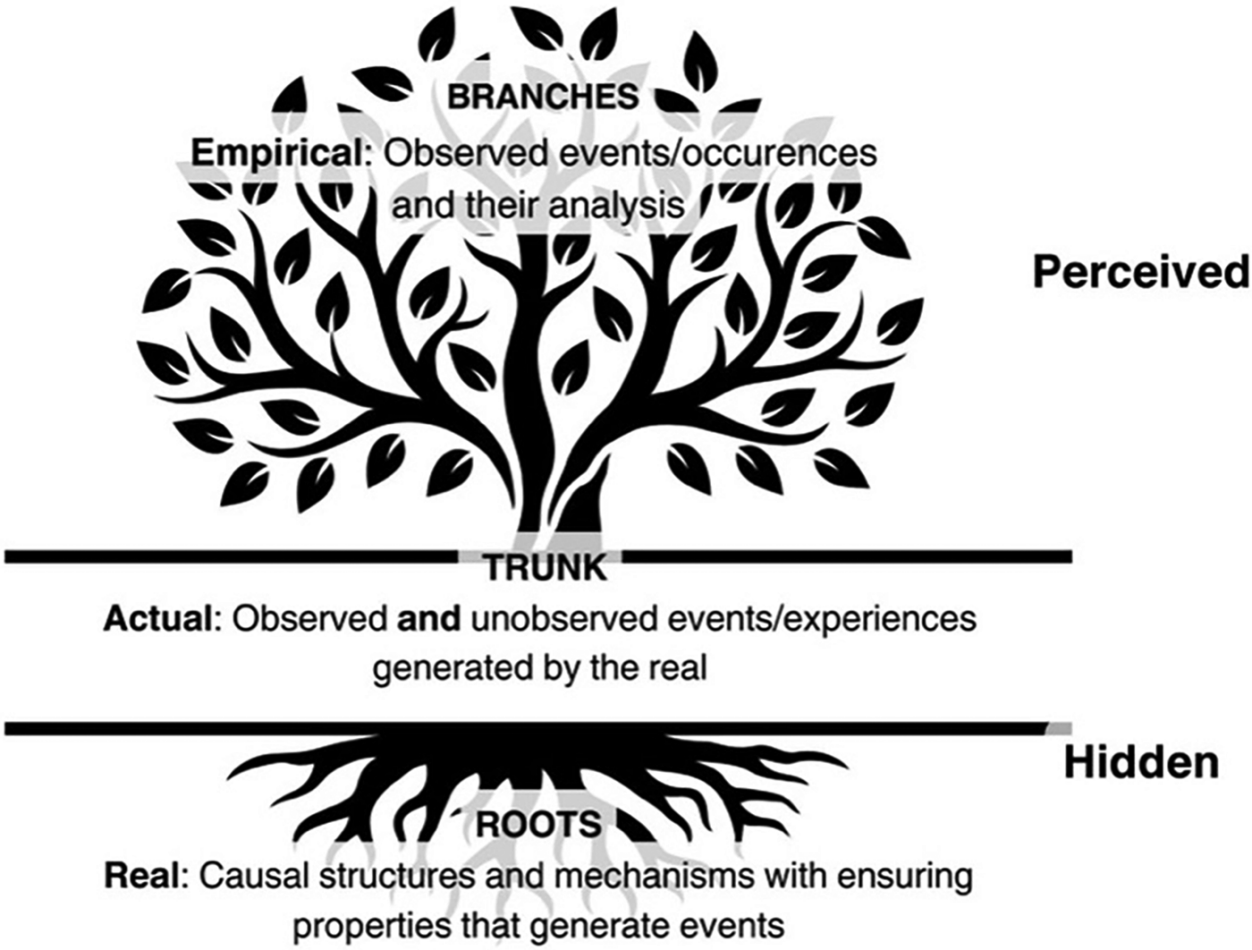
Illustration of a tree to describe the three ontological levels of critical realism: empirical, actual, and real.

The real level delves into the underlying mechanisms that drive the events and behaviors at the empirical and actual levels. This level reveals the generative structures like systemic racism, socioeconomic inequalities, and cultural influences that shape Black women's health decisions. It examines the deeper, often hidden, societal and policy-related factors—such as discriminatory healthcare practices, economic challenges, and prevailing cultural norms—that create barriers to PrEP uptake. At this level, critical realism seeks to uncover the root causes that need addressing to create meaningful change (Figure 1).

Understanding these three levels is crucial for developing effective PrEP implementation strategies for Southern Black women. It compels us to recognize that addressing the empirical evidence of low PrEP uptake requires interventions at the actual and real levels, targeting both the visible and invisible barriers. Such a multifaceted approach grounded in critical realism promises to not only bridge the gap between need and utilization but also transform the structural and cultural landscapes that currently inhibit the adoption of PrEP among Black women in the South.

## Results

### The empirical domain: observations and measured factors

This domain involves what we can directly observe and measure — the quantifiable interest levels in PrEP among Black women. Empirical evidence derived from the 15 articles demonstrates that southern Black women were largely unaware of PrEP; studies found that merely 10.7% of women had ever heard of PrEP (26).

#### Prep utilization

Data further reveals a spectrum of interest in PrEP utilization among Black women in the Southern U.S. The findings reveal significant variability in interest levels, with the maximum reported interest reaching 84.9%, indicating a strong potential for PrEP acceptance within this demographic (27). Similarly, a study found that after a brief description of PrEP, 173 (76.9%) expressed willingness to consider using PrEP, including 63 (87.5%) who were PrEP eligible, and another study found that 60.7% of southern Black women would be likely or very likely to use PrEP if available (28). Contrastingly, other studies illustrate a more tempered interest, such as one among 795 southern Black women in which 29.6% reported willingness to use PrEP, 35.6% reported unwillingness to use PrEP, and 34.8% were unsure about PrEP use (29). In a smaller sample of 27 southern Black women, 59.3% of focus group participants reported interest in PrEP (26). This empirical variability underscores an important illustration of PrEP acceptance among Black women, highlighting that while a majority exhibit a general willingness to consider PrEP, the degrees of interest are markedly heterogeneous across different segments of this population.

### The actual domain: reported experiences and unobserved events

The actual domain captures the events, including reported experiences, that may not have been directly observed. In examining the landscape of PrEP implementation among Black women in the Southern U.S., actual experiences illuminate the multifaceted barriers to uptake. Central to these experiences is the misalignment of marketing strategies, with awareness campaigns historically centered on gay, bisexual, and other men who have sex with men, rendering Black women virtually invisible (30–32). This misdirected focus has fostered a tangible sense of exclusion and frustration among Black women, underscoring the pervasive influence of racism and stereotypes resulting in over-targeting one community and potentially stigmatizing all Black communities, which obscures their access to critical HIV prevention methods.

#### Structural violence

Structural violence manifested through poverty, underemployment, and inadequate housing further compounds these barriers, creating an environment where prioritizing health and PrEP initiation becomes exceedingly challenging (33). These actual conditions reflect a direct impediment to accessing PrEP, entangled with the financial constraints that many Black women face. The financial burden associated with PrEP, encompassing medication costs, potential co-pays, and related healthcare expenses, emerges as a formidable obstacle, dissuading interest and initiation (32, 34–36).

#### Medical mistrust

Compounding these economic and structural barriers is a profound mistrust in the medical establishment (8, 32, 35). Concerns over pharmaceutical companies’ profit motives and historical injustices perpetrate a deep-seated skepticism towards biomedical interventions, including PrEP (8, 32). This mistrust is not unfounded but rooted in a lengthy history of medical racism, rendering the skepticism an actual barrier to PrEP acceptability.

#### Interpersonal dynamics

Interpersonal dynamics, particularly within sexual and romantic relationships, play a significant role in decision-making processes regarding PrEP. Fears of negative reactions, accusations of infidelity, and the potential for exacerbating intimate partner violence significantly deter interest in PrEP (32, 37), highlighting the gendered power imbalances that restrict Black women's autonomy over their health decisions. Stigma, related to HIV, perceived promiscuity and spiritual and cultural and Southern influences of residing in the “Bible belt” (8, 32, 33, 38), amplifies these challenges, creating a social environment where the decision to initiate PrEP is fraught with potential for judgment and misunderstanding.

#### Trusted providers

To further complicate this context, despite some evidence of a lack of trust in healthcare systems, southern Black women would prefer to receive PrEP from trusted primary healthcare providers (30, 31, 33–35). However, healthcare providers must be aware of and navigate implicit bias and stigma that act as a barrier to prescribing PrEP to adolescent and young adult Black women (39).

#### Awareness and knowledge

At the heart of these experiences lies a critical gap in PrEP awareness and knowledge (8, 27, 32, 36). Despite varying levels of awareness, a consistent theme is the inadequacy of accurate information about PrEP's purpose and intended use, with some misconceptions relegating it solely to use by men who have sex with men (30–32, 36). Moreover, the discordance between perceived and actual risk of HIV infection further complicates the landscape, with low-risk perception (30, 38) acting as a significant barrier to considering PrEP as a viable prevention strategy.

The actual experiences of Black women in the South, marked by structural, financial, interpersonal, and informational barriers, provide a rich tapestry of the lived realities that inhibit PrEP uptake. Often overshadowed or unacknowledged, these narratives are critical to understanding the complexities of PrEP implementation in this community. Addressing these barriers requires a multifaceted approach that not only acknowledges but actively engages with the lived experiences of Black women, fostering strategies that are responsive to their specific needs and challenges in the context of PrEP uptake.

### The real domain: tracing the evidence to the underlying mechanisms and structures

The real domain concerns underlying causal mechanisms that lead to the observed and unobserved events, such as socioeconomic factors and systemic issues. In the realm of the “real,” critical examination unveils the foundational causes and systemic conditions that hinder PrEP uptake among Black women in the South, transcending observable behaviors and individual-level barriers. At this level, gendered racism and classism emerge as core mechanisms, shaping a healthcare landscape where Black women are systematically marginalized. This exclusion is manifest in a broader societal narrative that devalues the health and well-being of Black women and impacts all aspects of their ability to attain PrEP services.

Systemic discrimination and underrepresentation*.* The poverty and economic barriers seen in the actual domain are symptoms of deeper, institutionalized racism and classism. This generative mechanism actively shapes Black women's access to resources, including PrEP. As evidenced by socioeconomic disparities such as poverty, underemployment, and inadequate housing, it serves as a backdrop against which the struggle for PrEP uptake unfolds (40–43). These conditions, deeply entrenched in historical inequalities and systemic discrimination, create an environment where accessing healthcare, including PrEP, becomes a secondary concern, overshadowed by the daily realities of survival and stability. The cost of PrEP, far from being a mere financial consideration, is intertwined with these socioeconomic challenges, further complicating access and initiation for those who might benefit most.

Underrepresentation and miscommunication in Public Health messaging. Public health campaigns and HIV prevention efforts have historically centered on populations other than Black women (e.g., men who have sex with men), creating a narrative that PrEP is not for them (44, 45). This underrepresentation and miscommunication lead to a lack of tailored information that aligns with Black women's lived experiences and social realities (46). This mechanism shapes the way Black women perceive their own HIV risk because they do not see themselves represented in the messaging. Consequently, they may believe that HIV prevention strategies like PrEP are not relevant to their circumstances, reinforcing a low perceived risk despite the actual prevalence of HIV within their communities.

Historical and contemporary medical gendered racism among Black women is a significant barrier to PrEP use that is rooted in a long history of medical exploitation and racism (47–52). This deep-seated skepticism towards healthcare institutions and pharmaceutical companies reflects a rational response to centuries of mistreatment and exploitation, highlighting the need for a profound transformation in how healthcare is delivered and communicated to marginalized communities. The stigma surrounding HIV and PrEP is deeply tied to cultural narratives and structural discrimination that have historically marginalized Black women's health needs. These narratives often frame HIV as associated with immoral behavior or promiscuity, reinforcing stigma against those who seek HIV prevention services. This stigma, fueled by structural racism and gendered discrimination, not only discourages Black women from perceiving their own vulnerability but also deters them from accessing PrEP for fear of being judged or labeled. The historical distrust of healthcare systems due to past mistreatment and exploitation further amplifies these stigmatizing attitudes and discourages engagement with PrEP.

These mechanisms reveal that the issues of low perceived HIV risk and stigma are not simply about individual behavior; they are deeply embedded in the way health systems and societal structures have historically communicated about and responded to Black women's health needs. Addressing these requires systemic change in public health messaging and efforts to dismantle stigma through culturally responsive care and education.

Gendered power structures reflect how societal norms and power imbalances restrict Black women's health autonomy and decision-making, shaping their interactions and choices regarding PrEP. Interpersonal dynamics, including the influence of sexual and romantic partners on PrEP decision-making, are not merely individual considerations but are reflective of these broader gendered power imbalances and societal norms that limit women's autonomy over their health decisions (52–57). This imbalance often manifests as interpersonal violence, where the threat or occurrence of violence from partners becomes a significant barrier for Black women when considering PrEP uptake. Concerns about potential backlash, accusations of infidelity, or even physical harm deter women from initiating discussions about PrEP with their partners, reinforcing the control and surveillance over their bodies and health choices (58–61). Addressing these intertwined issues requires a comprehensive strategy that not only challenges gendered power structures but also promotes safe environments where women can exercise autonomy over their health decisions without fear of violence or stigma.

At the “real” level, the challenges to PrEP uptake among Black women in the South are revealed to be complex and multi-layered, deeply embedded in the fabric of societal structures and historical and contemporary injustices. Addressing these challenges requires acknowledging these underlying mechanisms and committing to systemic change that prioritizes equity, trust, and inclusivity in healthcare provision and public health initiatives. Only by confronting these foundational issues can efforts to increase PrEP uptake among Black women in the South move from merely addressing symptoms to fostering meaningful and lasting change.

The synthesis of findings across the empirical, actual, and real domains illuminates the multifaceted nature of PrEP uptake barriers for Black women in the South. Empirical data on interest levels reflect an actual willingness affected by immediate barriers such as cost and partner influence. Yet, these are underpinned by real mechanisms, including structural violence, targeted marketing failures, systemic medical mistrust, and the pervasive stigma rooted in sociocultural narratives. This layered analysis underscores the need for multifaceted strategies that address surface-level barriers and the deeper structural and socio-cultural determinants to improve PrEP implementation in these communities effectively.

## Discussion

### The path forward

PrEP is the innovation, not the intervention. Implementation research commonly situates PrEP as the evidence-based intervention to prevent HIV; this positioning has vastly misaligned PrEP uptake strategies and barriers. Our initial recommendation is that the field reposition PrEP as the treatment/innovation and continue identifying interventions and strategies to deliver this treatment effectively.

The HIV biomedical prevention field is in a burgeoning moment of discovery, where burdensome daily oral PrEP regimens could become replaced with long-acting modalities affording superior compliance, greater effectiveness, and likely greater consumer desirability. However, the true underpinning of the current U.S. HIV epidemic is the contextual socio-political barriers that limit equitable service access to Southern Black communities. Without truly interrogating mechanisms that are driving disparities in PrEP utilization among priority populations, like Black women, population health gains will not be achieved in ending the HIV epidemic. Ultimately, these mechanisms have been in place and shaped the healthcare landscape over centuries; therefore, this review allows a detailed, systematic approach to understanding the complex reasons for critical gaps in PrEP coverage for Southern Black women. Without an emphasis on understanding mechanisms driving inequities and creating effective interventions to improve PrEP service delivery for Black Southern women, there is a high possibility of the further failed implementation of long-acting PrEP modalities due to cross-cutting, pervasive gendered racism, and classism that impacts the real-world setting in which Black women experience sexual health in the South.

Given the gaps in oral PrEP awareness and the need to optimize PrEP for Black women that we have described, we recommend numerous action strategies. For healthcare providers, they need to proactively incorporate sexual health education and discussions about HIV and PrEP into routine care for Black women. Facilitating provider-initiated education efforts, healthcare systems need flexibility to support longer appointments and a wider variety of clinical care appointments that would encompass education and preventive care. Outside of the healthcare setting, empowered community leaders can proudly be champions of PrEP use among Black women and normalize conversations about sexual health among their circles of influence. Researchers need to identify optimal strategies to disseminate comprehensive sexual health education that results in greater uptake of PrEP and other preventive reproductive health services (e.g., HIV/STI testing, cervical cancer screening, and vaccination, and family planning).

Addressing interpersonal dynamics and violence is critical, as it significantly impacts Black women's ability to access and adhere to PrEP. IPV can create unsafe environments, making discussions about sexual health or seeking prevention methods like PrEP difficult or dangerous. Thus, strategies must develop trauma-informed interventions that integrate IPV screening and support into PrEP services. While PrEP can be used discreetly, interventions should also empower Black women to navigate conversations with male partners, enhancing their confidence (62). Providing safe, confidential, and supportive spaces is essential for increasing PrEP uptake and sustained engagement among women experiencing IPV.

Finally, policymakers need to work to remove legal barriers to sexual health education in schools and codify laws that facilitate full access to comprehensive sexual health services (63, 64). Through numerous lenses and specializations, and by keeping Black women and their experiences centered, it is possible to increase knowledge about PrEP, yielding greater uptake during moments when exposure to HIV is most likely.

## Conclusion

This review underscores the urgent need to apply a critical realism framework to uncover the underlying causal mechanisms driving disparities in PrEP uptake and HIV incidence rates among Black women in the Southern United States. By delving into these unaddressed mechanisms, we have illuminated the complex barriers impeding PrEP implementation, highlighting the necessity for a comprehensive, community-centered approach. The findings of this review call for a concerted effort from healthcare providers, policymakers, community leaders, and researchers to dismantle systemic barriers, promote equitable access, and create environments that support informed health choices. Addressing these deep-seated issues through culturally competent care, robust sexual health education, and policy reforms is essential to ensure PrEP reaches those most in need. Only through such holistic and inclusive strategies can we transform the promise of PrEP into a tangible reality for Black women in the South, ultimately advancing health equity and moving closer to ending the HIV epidemic.
